# Synthesis of a Bone-Targeted Bortezomib with In Vivo Anti-Myeloma Effects in Mice

**DOI:** 10.3390/pharmaceutics10030154

**Published:** 2018-09-10

**Authors:** Hua Wang, Lifeng Xiao, Jianguo Tao, Venkat Srinivasan, Brendan F. Boyce, Frank H. Ebetino, Babatunde O. Oyajobi, Robert K. Boeckman, Lianping Xing

**Affiliations:** 1 Department of Pathology and Laboratory Medicine, University of Rochester Medical Center, Box 626, 601 Elmwood Ave, Rochester, NY 14642, USA; huawang@njmu.edu.cn (H.W.); Jianguo_Tao@URMC.Rochester.edu (J.T.); Brendan_Boyce@URMC.Rochester.edu (B.F.B.); 2 Institute of Stomatology, Nanjing Medical University, Jiangsu Key Laboratory of Oral Diseases, Nanjing 210029, China; 3 Department of Chemistry, University of Rochester, P.O. Box 270216, Rochester, NY 14627-0216, USA; lxiao2@ur.rochester.edu (L.X.); Srinivasan@URMC.Rochester.edu (V.S.); febetino@UR.Rochester.edu (F.H.E.); 4 Center for Musculoskeletal Research, University of Rochester, Rochester, NY 14627-0216, USA; 5 BioVinc, Pasadena, CA 91107, USA; 6 Department of Cell Systems & Anatomy, Mays Cancer Center, University of Texas Health Science Center at San Antonio, San Antonio, TX 78229, USA; oyajobi@uthscsa.edu

**Keywords:** drug delivery, bortezomib, velcade, bone targeting, multiple myeloma

## Abstract

Multiple myeloma (MM) is the most common cancer affecting the bone and bone marrow and remains incurable for most patients; novel therapies are therefore needed. Bortezomib (Btz) is an FDA-approved drug for the treatment of patients with MM. However, its severe side effects require a dose reduction or the potential discontinuation of treatment. To overcome this limitation, we conjugated Btz to a bisphosphonate (BP) residue lacking anti-osteoclastic activity using a novel chemical linker and generated a new bone-targeted Btz-based (BP-Btz) proteasome inhibitor. We demonstrated that BP-Btz, but not Btz, bound to bone slices and inhibited the growth of MM cells in vitro. In a mouse model of MM, BP-Btz more effectively reduced tumor burden and bone loss with less systemic side effects than Btz. Thus, BP-Btz may represent a novel therapeutic approach to treat patients with MM.

## 1. Introduction

Multiple myeloma (MM) is a plasma cell malignancy and the 2nd most common adult hematologic malignancy in the US. The 5-year survival rate is ~45% [1], and the disease remains incurable. Myeloma cells originate in the bone marrow (BM) where they destroy healthy BM cells, and eventually cause osteolytic lesions, which lead to bone pain and fractures. Current treatment for MM includes drugs, radiotherapy and eventually bone marrow transplantation, with chemotherapy being the first line treatment for newly diagnosed patients. Medication typically consists of 3 classes of drugs, including chemotherapy drugs that kill rapidly growing cells non-specifically, proteasome inhibitors that mainly target cancer cells, and corticosteroids that control inflammation caused by chemotherapy [2,3].

Bortezomib (Btz, marketed as VELCADE) is the first proteasome inhibitor used to treat MM and mantle cell lymphoma. It induces myeloma cell apoptosis by promoting excessive protein accumulation [4]. Myeloma cells are much more sensitive to Btz-induced cell death than normal or other types of cancer cells because they produce large amounts of immunoglobulin that induces a cell stress-unfolded protein response [5]. Btz prolongs the unfolded protein response by inhibiting proteasomal degradation of unfolded proteins, leading to cell cycle arrest and apoptosis [5,6,7]. Btz is given by intravenous or subcutaneous injection. It goes to all tissues of the body and often causes off-target effects, such as peripheral neuropathy and thrombocytopenia [8], which limits its utility or prevents the administration of effective doses. Because myeloma cells initially develop in the BM, delivering anti-MM drugs to the bone is an attractive approach to increase local drug concentrations and to reduce adverse effects on non-bone tissues.

The concept of bone-targeting pharmaceutical agents derives from bisphosphonates (BPs) based on their high binding affinity for hydroxyapatite in the bone matrix and their therapeutic efficacy in the treatment of bone disorders characterized by increased bone resorption. Bone-targeted BP conjugates have a high propensity to localize to the bone in vivo, typically at sites of active bone remodeling where the mineral component of the bone is exposed. As such, targeting small molecules to the bone offers the potential for significantly modifying the bio-distribution, both at the cellular level and in vivo. BPs with high bone-binding activity and minimal pharmacological activities should therefore be ideal carriers to deliver desired drugs to the bone [9]. In 2013, Agyin et al. synthesized several permanently linked BP-conjugated proteasome inhibitors, including Btz, and demonstrated that these conjugates killed MM cells in vitro at a similar dose range as their non-bone targeted counterparts [10]. However, no information has been reported as to whether these conjugates bind to the bone and provide any anticipated beneficial effect in vivo.

In this study, we designed and synthesized a new form of bone-targeted Btz (referred to as BP-Btz) by linking it to a BP residue using a boronate ester that was designed to release Btz from the BP at remodeling bone surfaces. We demonstrated, in a mouse model of MM, that BP-Btz binds to bone slices and has sustained and better efficacy than Btz.

## 2. Materials and Methods

### 2.1. Preparation of BP-Btz

#### 2.1.1. 2-(bis(2-hydroxyethyl)amino)ethyl (bis(diethoxyphosphoryl)methyl)carbamate (BP-Linker Tetraester)

A dry 10 mL round bottom flask at 0 °C under Ar was charged with 200 mg of tetraethyl amino- methylenediphosphonate (BP-tetraester) (0.66 mmol, 1 equiv), and triphosgene (78 mg, 0.264 mmol, 0.4 equiv) in 3 mL of dry CH_2_Cl_2_. Triethylamine, (67 mg, 0.66 mmol, 1 equiv) was added to the reaction mixture. The resulting reaction mixture was allowed to warm to rt and stir overnight. The reaction mixture was quenched by the addition of 25 mL of 1 M aq HCl. After the separation of the phases, the aqueous layer was extracted with CH_2_Cl_2_ (3 × 30 mL). The combined organic layers were washed with sat aq NaCl solution (15 mL), dried over Na_2_SO_4_, filtered, and concentrated under vacuum to obtain a yellow oil (190 mg). A dry 5 mL round bottom flask was charged with the preceding crude product mixture (190 mg), followed by dibutyltin dilaurate (DBTDL) (36.6 mg, 0.058 mmol, 0.1 equiv), and 77.5 mg (0.52 mmol, 0.9 equiv) triethanolamine (TEOA) in 1.2 mL of dry CH_2_Cl_2_. The resulting reaction mixture was allowed to stir at rt for 2 days. The reaction mixture was then filtered through a celite pad and the pad was washed with additional CH_2_Cl_2_ (15 mL). The filtrate was concentrated under vacuum, and the residue was purified by column chromatography (CH_3_OH-CH_2_Cl_2_ gradient, 5:95 to 2:8) to afford 126 mg (50%) of the BP-Linker-Tetraester as a colorless oil. R_f_ = 0.45 (CH_3_OH-CH_2_Cl_2_, 2:8); KMnO_4_ active.

^1^H NMR (500 MHz, D_6_ DMSO): δ 8.21 (d, *J* = 10 Hz, 1H), 5.27 (s, 2H), 4.43–4.32 (m, 3H), 4.07 (s, 9H), 3.48 (s, 5H), 3.28 (s, 4H), 1.23 (m, 13H) ^13^C NMR (400 MHz, CDCl_3_): δ 115.36, 63.98, 59.63, 56.64, 56.03, 53.68, 46.17 (t), 16.37 ^31^P NMR (400 MHz, CDCl_3_): δ 13.085. IR cm^−1^: 3427, 2981, 2927, 1714, 1537. HRMS (ESI) *m*/*z* (M + H) Calcd for C_16_H_37_N_2_O_10_P_2_: 479.1917. Found: 479.1923.

#### 2.1.2. (((2-(8-((R)-3-methyl-1-((S)-3-phenyl-2-(pyrazine-2-carboxamido)propanamido)butyl)tetrahydro-8H-4I4,8I4-[1,3,2]oxazaborolo[2,3-b]-[1,3,2]oxazaborol-4-yl)ethoxy)carbonyl)amino)methylene)bis(phosphonic acid (BP-Btz)

A dry 10 mL round bottom flask was charged with pre-activated (at 120 °C) powdered 3Å molecular sieves (1 g) and a solution of BP-Linker-Tetraester (125 mg, 0.26 mmol) and (120.5 mg of Bortezomib (Btz) (0.31 mmol, 1.2 equiv) in dry THF (5 mL). The reaction mixture was stirred at rt for 3 days. The mixture was filtered through glass wood, then a celite pad, and concentrated in vacuo. The crude product was washed with Et_2_O (10 mL × 2) to remove the unreacted Btz affording 215 mg of crude product as a brown solid. Crude material was characterized by mass spectroscopy and exhibited no ion at *m*/*z* 385.2 corresponding to Btz: Thermo-MS (ESI) *m*/*z* (M + H) Calcd for C_35_H_58_BN_6_O_12_P_2_: 827.3. Found: 827.4. *m*/*z* (M + Na) Calcd for C_35_H_57_BN_6_O_12_P_2_Na: 849.35. Found: 849.4. *m*/*z* (M + K) Calcd for C_35_H_57_BN_6_O_12_P_2_K: 865.32. Found: 865.3. A dry 10 mL round bottom flask was charged with a solution of the above crude product (215 mg = 26 mmol) in dry CH_2_Cl_2_ (8 mL) under Ar at 0 °C. Trimethylsilyl bromide (TMSBr) (239.7 mg, 0.156 mmol, 6 equiv) was added dropwise with magnetic stirring. After the addition was complete, the reaction mixture was allowed to stir and warm to rt overnight. The reaction mixture was then concentrated in vacuo and kept under high vacuum for 10 min to afford a brown solid. Then, methanol (5 mL) was added to dissolve the solid, and the resulting solution was concentrated in vacuo. This procedure was repeated 3 additional times. The resulting solid was dissolved in EtOH (0.5 mL), and this was followed by the addition of 10 mL of Et_2_O, resulting in the formation of a brownish-white precipitate. The solid was isolated by filtration and dried under high vacuum at 90 °C affording 170 mg of **3** (BP-Btz) as a light brown solid (91%). Characterization Data for BP-Btz: ^1^H NMR (500 MHz, CDCl_3_): δ 8.95 (s, 1H), 8.68 (s, 1H), 8.62 (s, 1H), 7.37 (s, 4H), 7.16 (s, 1H), 4.71 (t, *J* = 10 Hz, 1H), 4.30 (s, 2H), 3.94 (t, *J* = 20 Hz, 1H), 3.74 (s, 4H), 3.47 (s, 2H), 3.28 (s, 4H), 3.08 (m, 1H), 2.97 (m, 1H), 2.78 (m, 1H), 1.32–1.24 (m, 2H), 1.16 (m, 1H), 0.72 (m, 6H). ^13^C NMR (400 MHz, CDCl_3_): δ 177.38, 163.85, 155.7, 148.34, 144.34, 144.14, 143.88, 135.23, 129.72, 128.77, 127.31, 59.59, 55.81, 55.68, 52.67, 50.67, 48.74, 47.88, 36.39, 24.83, 23.85, 21.99. ^31^P NMR (400 MHz, CDCl_3_): δ −1.43, −4.81. IR cm^−1^: 3275, 3055, 2954, 2146, 1716, 1701, 1676, 1629, 1521. TOF-MS (ES+) *m*/*z* (M + 3H) Calcd for C_27_H_44_BN_6_O_12_P_2_: 717.26. Found: 717.55. *m*/*z* (M + 2H + Na) Calcd for C_27_H_43_BN_6_O_12_P_2_Na: 739.24. Found: 739.64. *m*/*z* (M + H + 2Na) Calcd for C_27_H_42_BN_6_O_12_P_2_Na_2_: 761.22. Found: 761.59. HRMS (ESI) *m*/*z* (M + Na) Calcd for C_27_H_41_BN_6_O_12_P_2_Na: 737.2241. Found: 737.2248.

#### 2.1.3. 2-(bis(2-hydroxyethyl)amino)ethyl (bis(dihydroxyphosphoryl)methyl)carbamate (1 BP-Linker)

An oven dried round bottom flask was charged with a solution of 0.1 g of 2-(bis(2-hydroxyethyl)amino)ethyl(bis(diethoxyphosphoryl)methyl)carbamate (BP-Linker-) (0.209 mmol) in 1 mL anh CH_2_Cl_2_. After cooling to 0 °C, neat bromotrimethyl silane (0.17 mL, 1.25 mmol) was added dropwise and the content was gradually warmed to room temperature and stirred for 16 h. The reaction mixture was concentrated under reduced pressure and treated with 5 mL of anh methanol. The alcoholic solution was concentrated and the procedure was repeated three times to yield a pale brown waxy solid. The crude mixture was dissolved in a methanol/water mixture and added to diethyl ether, whereupon 47 mg (62%) of 2-(((diphosphonomethyl)carbamoyl)oxy)*N*,*N*-bis(2-hydroxyethyl) ethan-1-amin- ium bromide (BP-Linker) precipitated as a white waxy solid.

Characterization data for BP-Linker: ^1^H-NMR (400 MHz, CD_3_OD/D_2_O) δ 4.59–4.41 (m, 2H), 4.30 (t, *J* = 2.4 Hz, 1H), 4.03–3.89 (m, 4H), 3.72–3.61 (m, 2H), 3.59–3.41 (m, 4H); ^31^P-NMR (400 MHz, CD_3_OD/D_2_O) δ 14.99; ^13^C-NMR (100 MHz, D_2_O) δ 156.69, 59.49, 55.38, 55.02, 52.87, 48.36 (t, *J* = 137 Hz).

### 2.2. Animal Experiments

6-week-old female NIH-III nude (NIH-Lyst^bg-J^Foxn1^nu^Btk^xid^) were purchased from Charles River (Wilmington, MA, USA). 5TGM1-GFP mouse myeloma cells have been described in detail by Babatunde O. Oyajobi (University of Texas Health Science Center at San Antonio, San Antonio, TX, USA) [11,12,13]. The mice received 5TGM1-GFP MM cells (0.5 × 10^6^ in 10 microliter volume) via a tail vein injection. The mice were randomized into PBS Veh, Btz and BP-Btz groups (*n* = 6–8 mice/group) based on body weight on day 1 after the tumor cell inoculation and were treated with the molar equivalent dose of Btz (0.6 mg/kg, intraperitoneal injection) and BP-Btz (1.2 mg/kg) 3 times/week for 2 weeks. The drugs were dissolved in DMSO to make 50 mM stock solution and were then diluted with PBS. The mice were sacrificed one week later (at 3 weeks post-tumor cell injection). The end-point outcome measures included serum IgG2b levels, tumor burden, bone volume, routine blood counts and osteoblast differentiation. A group of mice with no tumor cell injection were used as control mice. All animal use in this study has been approved by the Animal Care and Use Committee at the University of Rochester (UCAR#2001-121, 100661).

#### 2.2.1. Enzyme Linked Immunosorbent Assay (ELISA)

Blood was collected by a facial vein puncture on the day when the mice were sacrificed. The serum was stored at −80 °C. The levels of 5TGM1-specific monoclonal paraprotein (IgG2b kappa) were assayed using a specific ELISA kit (IGG-1-2B, Life Diagnostics, West Chester, PA, USA).

#### 2.2.2. IVIS Imaging

Following the administration of an inhalation anesthetic, the mice were subjected to an IVIS^®^ SpectrumCT preclinical in vivo imaging system (PerkinElmer, Waltham, MA, USA) to measure GFP fluorescence before they were sacrificed. To support absolute quantitation, the system measures dark charge during down-time and runs a self-calibration during initialization. To start imaging, the IVIS system was initialized (one click) and the imaging parameters for the experiment were set. The GFP signal intensity in the tibia, whole body, and head was analyzed using IVIS spectrum imaging system software and presented as photons per second.

#### 2.2.3. MicroCT

Femurs were dissected free of soft tissue, fixed overnight in 10% buffered formalin, and scanned at high resolution (10.5 μm) on a VivaCT40 μCT scanner (Scanco Medical, Bassersdorf, Switzerland) using 300 ms integration time, 55 kVp energy, and 145 μA intensity. 3D images were generated using a constant threshold of 275 for all samples. Trabecular bone parameters were assessed, including BV/TV, Tb.Th, Tb.N and Tb.Sp.

#### 2.2.4. Histology and Histomorphometric Analysis

The legs including femora and tibiae were fixed in 10% buffered formalin, decalcified in 10% EDTA, and embedded in paraffin for sectioning or Tissue-Tek for frozen sectioning. (1) Paraffin sections (4 μm) were stained with H&E and converted to digital images using an Olympus VS120 whole slide imaging system (Olympus, Center Valley, PA, USA). Histomorphometric analyses were performed on sections cut at 3 representative levels in each bone. The percentage of trabecular bone (BV/TV) was analyzed using the automated algorithm we developed with the Visiopharm Image Analysis System [14]. (2) The frozen sections (8 μm) were cut using a Leica CM1850 cryostat (Leica, German) and mounted with a mounting medium containing DAPI (Vector Lab., Burlingame, CA, USA). The slides were scanned using the VS120 whole slide scanner. Analyses were performed on sections cut at 3 representative levels in each bone. GFP+ areas in tibiae and femora were analyzed using Image Pro-Plus version 6.0 software (Media Cybernetics, Rockville, MD, USA).

#### 2.2.5. Cell Cultures

(1) Bone binding assay. Bone slices were prepared from samples of cow femoral cortical bone that had been cut transversely into roughly 20 × 10 mm columns using a band saw. We cut 1.5 mm thick sections from the bone columns using a Buehler IsoMet Low Speed saw (Buehler, Lake Bluff, IL, USA) and then used a round 4.8 mm diameter hole puncher to make circular slices that could be dropped into wells in 96-well plates. Bone slices were pre-incubated with Veh, BP, Btz or BP-Btz for 24 h, washed extensively with PBS, and transferred to new culture plates. 5TGM1-GFP MM cells (3.2 × 10^4^ cells/slice) were seeded on drug pre-incubated bone slices for 24 h. The number of living cells was determined using a Cell Counting Kit-8 (Dojindo Molecular Technologies, Inc., Rockville, MD, USA). (2) Cell survival assays. 5TGM1-GFP MM cells were plated on 12-well plastic culture plates and treated with different amounts of testing agents for 24 h. The number of living cells was counted using a Trypan Blue exclusion method. (3) Osteoblast differentiation. Bone-derived stromal cells were generated using a recently published protocol [15]. Cells were cultured in 60-mm dishes at 2 × 10^6^ cells/dish in a α-MEM culture medium containing 10% FCS with or without 50 μg/mL ascorbic acid and 10 mM β-glycerophosphate. Media were changed every 4 days for 12 days. At the end of the culture period, cells were fixed in formalin and stained for Alkaline Phosphatase (ALP) activity. The ALP^+^ area was assessed.

#### 2.2.6. Routine Blood Counting

Blood was collected via retro-orbital sinus and stored in a microtainer (BD, 365974) for less than 1 h before analysis. Complete blood count values were acquired using 20–30 μL of whole blood using the scil Vet ABC Plus hematology analyzer (Scil Animal Care Company, Gurnee, IL, USA).

### 2.3. Statistical Analysis

All results are given as mean ± SD. The statistical analysis was performed using GraphPad Prism 5 software (GraphPad Software Inc., San Diego, CA, USA). Comparisons between the 2 groups were analyzed using the 2-tailed unpaired Student’s *t* test. One way ANOVA and Dunnett’s post-hoc multiple comparisons were used for comparisons among 3 or more groups. *p* values under 0.05 were considered statistically significant.

## 3. Results

**Figure 1:**
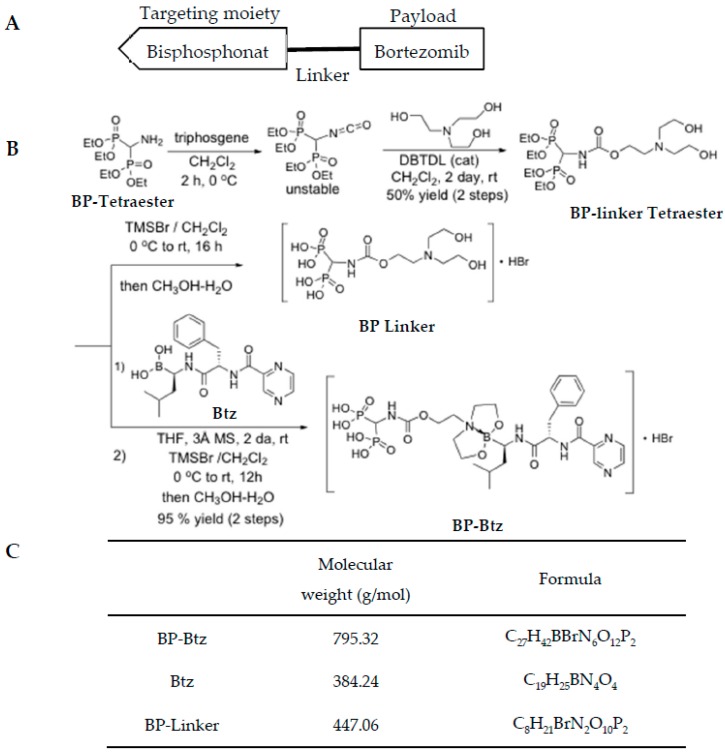
Generation of Bisphosphonate-Bortezomib (BP-Btz) conjugate. (**A**) Diagram showing the principle of our bone targeting strategy, including a bisphosphonate, a linker, and a payload that usually is a drug or other pharmacological inhibitor. (**B**) Steps of the BP-Btz synthesis. (**C**) Molecular weight and chemical formulation of BP-Btz, Btz or BP-linker.

Design and synthesize bone targeted Bortezomib. To generate a bone-targeted Btz, we selected a BP as a targeting moiety based on its high bone affinity property [16,17] (Figure 1A) and its potential to release this proteasome inhibitor at the bone surface due to the lower pH and enzymatic environment of high turnover sites. Because BPs have an anti-osteoclast activity that might directly affect cancer cell growth in the BM, we purposely chose a simple non-bioactive BP that has little effect on osteoclasts to avoid potential confusion on data interpretation. The synthesis of BP-Btz was outlined in Figure 1B. The design of the BP-Btz conjugate entailed the identification of a linker to which a biologically inactive bis-phosphonic acid, such as (aminomethylene)bisphosphonic acid, could be attached, leaving a diethanolamine fragment available to generate a 1,3,2-dioxazaboroyl fragment upon coupling with Btz. The dioxaborolyl release group was identified as having the necessary stability at pH 7.4 for transport to the bone but sufficient acid liability to be cleaved at the low pH (pH ≈ 4–5) present under the ruffled border of osteoclasts and release Btz. The synthesis of the BP-linker tetraester diol began with the generation of the intermediate isocyanate from tetraethyl (aminomethylene)bisphosponate upon treatment with triphosgene followed by the addition of triethanolamine and a catalytic amount of dibutyltin dilaurate (DBTDL) affording the diol (BP-linker tetraester) in a 50% yield over two steps, as shown in Figure 1B. The desired BP-Btz conjugate was then obtained by dioxazaboroxane formation from BP-linker tetraester and Btz boronic acid in methylene chloride in the presence of 3 Å molecular sieves over 3 days at room temperature (85–90% conversion, 95% yield based upon conversion), followed by the direct treatment of the crude solution of the BP-Btz tetraethyl esters with TMSBr. The removal of unreacted starting materials was possible via the precipitation of the BP-Btz conjugate from a mixture of ethanol and diethyl ether. Figure 1C shows the molecular weight and chemical formulation of BP-Btz, Btz, and BP-linker.

**Figure 2:**
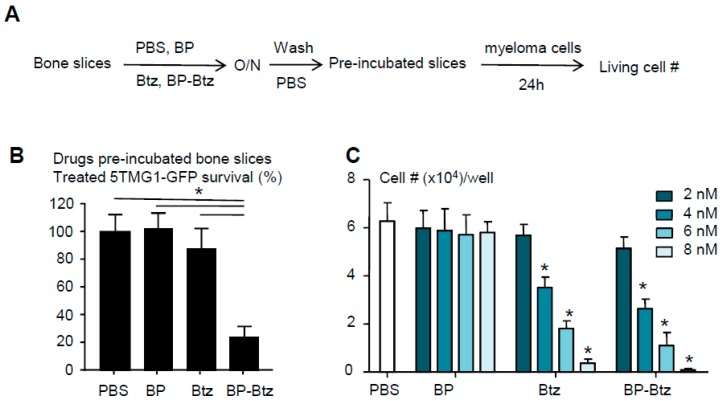
BP-Btz binds to bone matrix and maintains its bioactivity to kill myeloma cells. (**A**) Diagram showing the bone binding assay. (**B**) Bone slices were pre-incubated with PBS, Btz, BP-Btz overnight and then washed with PBS. 5TGM1-GFP myeloma cells were cultured with bone slices for 24 h and cells survival was measured using a Cell Counting Kit-8. Data are means ± SD of 6 wells. 3 experimental repeats. One way ANOVA with Dunnett’s test. * *p* < 0.05. (**C**) Cells were treated with different doses of drugs for 24 h. The number of living cells was counted using a Trypan Blue exclusion method. Data are means + SD. N = 4 wells. 3 experimental repeats. One way ANOVA with Dunnett’s test. * *p* < 0.05 vs. PBS.

BP-Btz binds to bone matrix and maintains its bioactivity to kill myeloma cells. Our overall hypothesis is that BP-Btz binds to the bone where Btz is released and exhibits its bioactivity locally. To test this, we developed a bone binding assay, in which we pre-incubated the bone slices with 1 μM BP-Btz, Btz or BP-linker overnight, removed the drug solutions, washed bone slices with PBS extensively, seeded MM cells onto the bone slices for 24 h, and then examined the surviving cell numbers on the bone slices (Figure 2A). The prediction is that MM cells will die off when they are seeded on the bone slices that are pre-incubated with BP-Btz, but will survive if they are seeded on the bone slices that are pre-incubated with Btz, BP-linker or PBS because the Btz or PBS will be washed away leaving no inhibitor on the slices, and because BP-linker itself has no anti-MM activity. As expected, we found that MM cells died when we cultured them on bone slices that had been pre-incubated with BP-Btz, but not when Btz or BP-linker was used (Figure 2B). We treated 5TGM1 cells with different doses of BP-Btz, Btz or BP-linker and demonstrated that BP-Btz and Btz had a similar efficacy to kill them in vitro while BP-linker had no such effect (Figure 2C).

**Figure 3:**
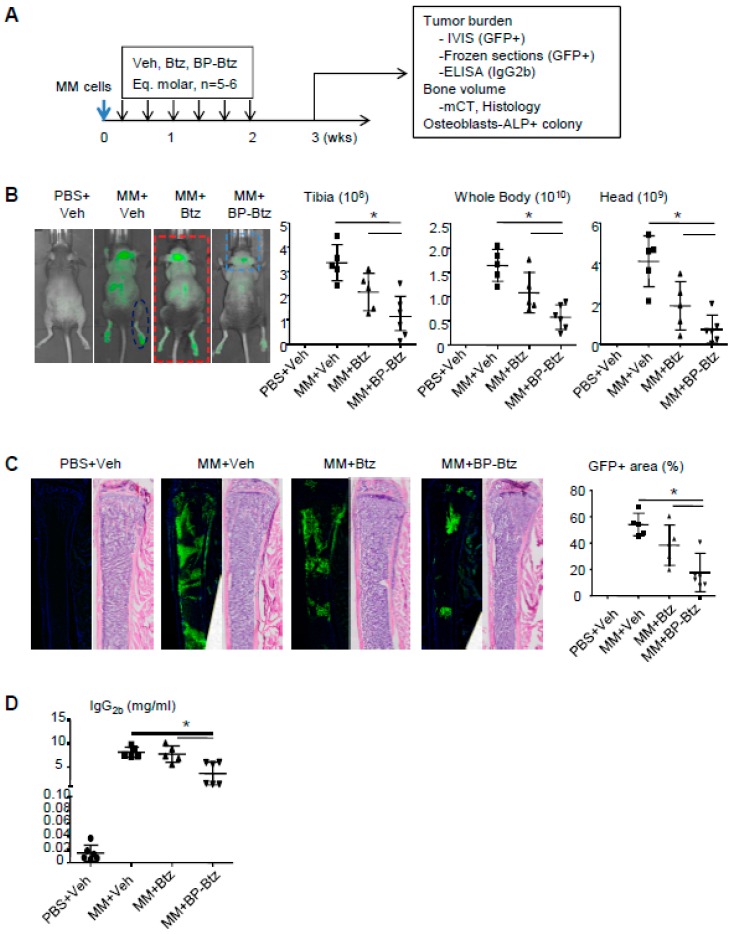
BP-Btz reduces tumor burden in a mouse model of MM. (**A**) Mice injected with 5TGM1-GFP cells were treated with Veh, Btz, or BP-Btz for 2 weeks and subjected to outcome measures 1 week post-treatment. (**B**) Distribution and signal intensity of GFP+ cells by IVIS imaging. (**C**) GFP+ area on frozen sections of femoral bone. (**D**) Serum levels of mouse IgG2b by ELISA. Data are means ±SD of 5–6 mice. One way ANOVA with Dunnett’s test for comparison of MM + Veh, MM + Btz, and MM + BP-Btz groups. * *p* < 0.05.

BP-Btz reduces the tumor burden and bone destruction more effectively than Btz in a mouse model of MM. Btz is a first line treatment for patients with MM. We hypothesized that BP-Btz would be more effective at reducing the tumor burden and myeloma-induced bone destruction than Btz in vivo. We used the 5TGM mouse model of MM, in which mouse 5TGM myeloma cells carry a green fluorescence protein (GFP) that allows the measurement of the distribution of GFP+ myeloma cells. In this model, myeloma cells entered the BM and spleen after tail vein administration [11]. We randomized the NIH-III nude mice into 4 groups based on their body weight (*n* = 6–8 mice/group). The mice in group 1 received a PBS injection as non-MM control. The mice in groups 2–4 received MM cells via the tail vein injection. The mice were treated one day post-MM cell inoculation with a saline vehicle (Veh), or the molar equivalent dose of Btz (0.6 mg/kg) and BP-Btz (1.2 mg/kg), once every 2 days for 2 weeks, and were sacrificed one week later as outlined in Figure 3A (Supplementary Table S1). In this study, we used a short dosing regimen to determine if BP-Btz has better efficacy than Btz to reduce the tumor burden. The tumor burden was measured as a GFP signal intensity by IVIS imaging, GFP+ area on frozen sections, and serum idiotypic IgG2b levels by ELISA. The MM-associated bone loss (bone volume) was assessed by μCT and by histomorphometric analysis on H&E-stained sections. The effect of MM cells on osteoblast function was assessed by an ALP+ colony forming assay using BM stromal cells from the mice. As expected, MM-bearing mice treated with Veh developed multiple tumor masses in the skeleton including the long bones, vertebrae, and skull, and in the spleen as indicated by the GFP intensity (Figure 3B and Supplementary Figure S1). Using this sub-optimal dosing regimen, Btz failed to significantly reduce the GFP intensity, while BP-Btz significantly decreased the GFP intensity (Figure 3B). This was further confirmed by measuring the GFP+ area on frozen sections (Figure 3C) and serum IgG2b levels (Figure 3D).

**Figure 4:**
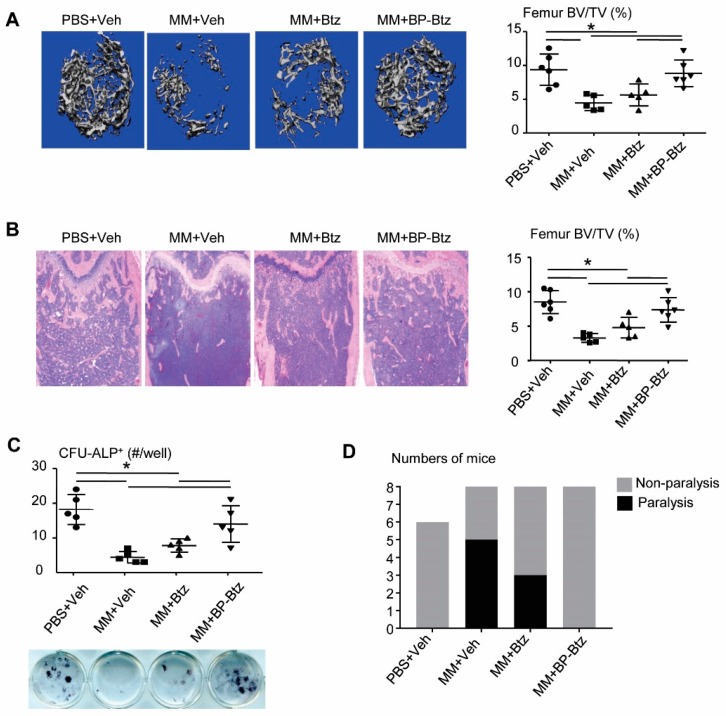
BP-Btz reduces bone loss and osteoblast inhibition in a mouse model of MM. Mice were described in Figure 3A. (**A**) Micro-CT images and data analysis of femoral bones. (**B**) Images of H&E-stained sections and femoral bone volume by histomorphometric analysis. (**C**) BM cells were cultured in the OB-inducing medium for 12 days. CFU-ALP+ colonies were identified by ALP staining. Data are means ± SD of 5–6 mice. One way ANOVA with Dunnett’s test for comparison of PBS + Veh, MM + Veh, MM + Btz, and MM + BP-Btz groups. * *p* < 0.05. (**D**) Number of paralyzed mice in each group.

MM develops primarily in the BM and it is associated with severe bone loss. Results from μCT (Figure 4A) and histomorphometric analysis (Figure 4B) revealed a marked bone loss in Veh-treated MM-bearing mice. Similar to the tumor burden shown in Figure 3B–D, Btz failed to prevent bone loss, while BP-Btz-treated mice had normal bone volume values, similar to the levels in non-MM-bearing mice. One of the mechanisms by which MM cells cause bone loss is to inhibit osteoblast differentiation [18]. Btz directly promotes osteoblast differentiation in the absence of MM cells [19]. Thus, it is likely that cells from BP-Btz-treated mice have a higher osteoblast differentiation potential than cells from Btz-treated mice because BP-Btz kills more MM cells and perhaps has stronger effects on osteoblast precursors due its delivery of a higher local concentration of Btz. To test this, we performed an ALP+ colony assay using BM stromal cells from Btz- or BP-Btz treated mice. Cells from Veh-treated MM mice had obviously reduced ALP+ colonies. Cells from BP-Btz-treated mice formed significantly more ALP+ colonies than cells from Btz-treated and Veh-treated MM mice (Figure 4C). Consistent with the above results, at the time the experiments were terminated all MM-bearing mice treated with Veh had developed paralysis, whereas there was a 40% decrease in the incidence of paralysis in tumor-bearing mice treated with Btz. Even more dramatically, none of the mice treated with BP-Btz exhibited paralysis (Figure 4D).

**Figure 5:**
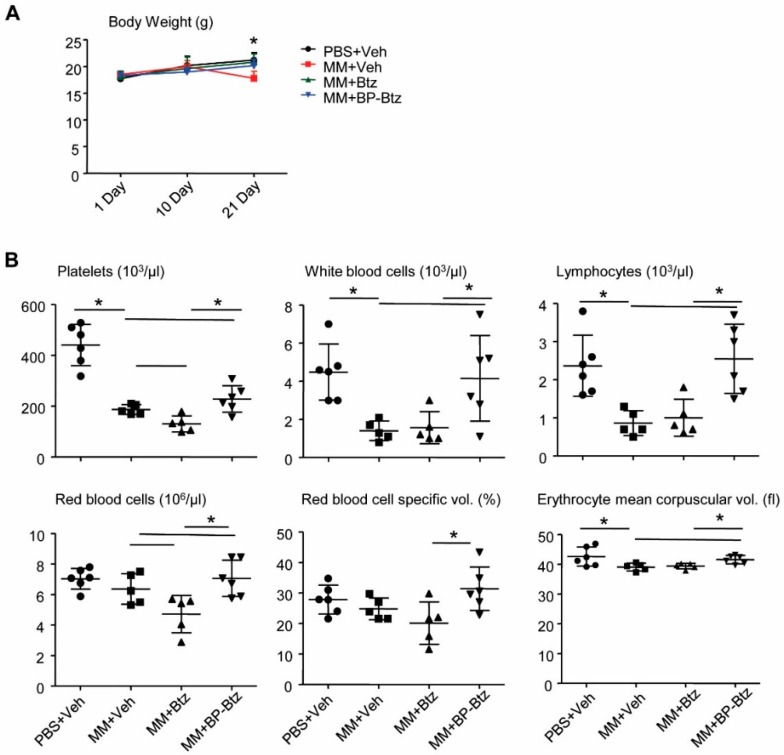
BP-Btz causes less severe pancytopenia in MM mice than Btz. Mice described in Figure 3A were used. (**A**) Body weight. (**B**) Routine blood testing was performed. Data are means ± SD of 5–6 mice. One way ANOVA with Dunnett’s test for comparison of PBS + Veh, MM + Veh, MM + Btz, and MM + BP-Btz groups. * *p* < 0.05.

BP-Btz induces less severe pancytopenia than Btz in MM mice. Pancytopenia is a common systemic side effect of anti-cancer drugs, including Btz. Specifically, thrombocytopenia is one of the major and commonly-observed Btz-related toxic effects seen in MM patients [20]. Because BP-Btz is designed to only deliver “free” Btz locally after the BP moiety binds to the bone and the Btz is released as a consequence of the local acidic environment, we anticipated that BP-Btz would cause less severe pancytopenia or thrombocytopenia than Btz. To test this, we compared body weights before and after the drug treatment and performed routine blood counts at the end of the experiment in MM-bearing mice described in Figure 3 and Figure 4. There was a small, but significant decrease in body weight in Veh-treated MM-bearing mice, but not in Btz- and BP-Btz-treated mice (Figure 5A). Compared to the control mice that had no-MM cell inoculation, Veh-treated MM-bearing mice had markedly decreased numbers of white blood cells and platelets. Btz-treated MM-bearing mice had a further reduction in platelet numbers. Impressively, BP-Btz-treated mice had white blood cell numbers similar to those in non-MM mice, and significantly higher numbers of platelets than both Veh- and Btz-treated MM-bearing mice. BP-Btz also had a similar beneficial effect on red blood cell parameters (Figure 5B).

## 4. Discussion

Btz is a first-line drug used to treat MM with high response rates in patients with both relapsed/refractory and newly diagnosed MM [21]. It acts as a proteasome inhibitor and reversibly inhibits the mammalian 26S proteasome [22]. Btz induces misfolded protein accumulation in myeloma cells followed by endoplasmic reticulum stress-associated apoptosis [23]. However, Btz has off–target adverse effects following its systemic administration, limiting the amount of drug that can be administered in patients. MM cells reside mainly in the BM and directly affect adjacent bones. BPs have a high bone-binding activity and can thus be used as carriers to deliver desired drugs to the bone [9]. In this study, we designed and synthesized a bone-targeted form of Btz and demonstrated for the first time that a bone-targeted Btz-based conjugate has a significant anti-MM effect in mice.

Bone-targeting anti-cancer drugs to the bone have been proposed for decades due to the potential benefits of lower systemic toxicity, lower dosage requirement and increased efficacy. For example, in 2008, scientists in ARIAD Pharmaceuticals, Inc., synthesized a bone-targeted Src tyrosine kinase inhibitor. We characterized it and demonstrated its anti-resorptive activity [24] and anti-tumor effects [25], but the lead compound was not developed into a drug. Btz is an ideal candidate for bone targeting because MM cells grow predominantly in the BM. In 2013, Agyin et al. made a BP (alendronate) linked Btz and other proteasome inhibitors and reported that these BP conjugates kill MM cells in a similar dose range to Btz [10]. However, the authors did not test if these BP-Btz conjugates bind to the bone, and more importantly if they have anti-cancer activity in vivo. Another issue is that they used an active BP residue (alendronate), potentially making it difficult to separate the BP’s effect from Btz’s effects on osteoclasts.

Our drug design has 2 features that distinguish it from previous published BP-Btz conjugates [10]. The first is that we used a form of BP that does not have an anti-resorptive activity, allowing us to test the effect of Btz alone. Another is that we used a chemical linker that is stable in the bloodstream, but is likely cleaved by hydrolysis in the acidic microenvironment under the osteoclast ruffled border in resorption sites to release the active drug from bone surfaces. We demonstrated that our BP-Btz bound to bone matrix and reduced MM burden and bone loss with higher efficacy than Btz, indicating that the bone-targeting of Btz does not affect its anti-cancer bioactivity.

This is the first description of a BP-Btz conjugate that exhibits an in vivo efficacy superior to Btz alone in a prevention protocol. We are currently pursuing an intervention regimen in similar mouse models carrying human MM cells. One of the clinical adverse effects of Btz is peripheral neuropathy, which is assessed primarily based on neurologic clinical examination and neurophysiologic methods [8]. Current knowledge of the mechanism underlying Btz-induced neurotoxicity is very limited [26]. Mitochondrial and endoplasmic reticulum damage seems to play a key role since Btz is able to activate the mitochondrial-based apoptotic pathway [27], which will be examined in future studies. The current paper focused on one example of BP-Btz conjugates; conjugates with different release rates may impact efficacy and are being evaluated. Furthermore, for bone-targeting purposes, to allow a clear separation of the bioactivity of the BP-Btz conjugate from any bioactivity of the BP, we used a BP without an anti-resorptive activity. In future studies, we can consider making drug conjugates with active BPs to determine if this increases efficacy without adverse BP effects. We are performing pharmacokinetic and pharmacodynamic studies to confirm that the Btz is indeed released on the bone from the BP-Btz conjugate. Finally, we only used a single dose of BP-Btz and have not performed the dose-limiting toxicity (DLT) study. It is therefore possible that DLT is higher (or lower) for the conjugate. Thus, a DLT study using different doses of BP-Btz will be needed to conclude that the BP-Btz is more effective that Btz.

In summary, we synthesized a new conjugate form of Btz (BP-Btz) and demonstrated its efficacy in vivo. Overall, BP-Btz may represent a novel therapeutic approach to treat patients with MM with overall improved efficacy and potentially less systemic adverse effects.

## Supplementary Materials

The following are available online at http://www.mdpi.com/1999-4923/10/3/154/s1, Figure S1: Mouse injected with 5TGM1-GFP cells via the tail vein and subjected to IVIS imaging at 3 weeks post-MM cell inoculation. The IVIS image from a MM mouse (right) showing GFP+ MM cells in skull, vertebrae, spleen and legs and from a mouse given no MM cells (left); Table S1: Dosing information of BP-Btz in MM mice.

## References

[1] San Miguel J., Blade J., Boccadoro M., Cavenagh J., Glasmacher A., Jagannath S., Lonial S., Orlowski R.Z., Sonneveld P., Ludwig H. (2006). A practical update on the use of bortezomib in the management of multiple myeloma. Oncologist.

[2] Shaji K., Kumar N.S.C. (2018). National Comprehensive Cancer Network (nccn) clinical practice guidelines in oncology. Multiple Myeloma.

[3] Dingli D., Ailawadhi S., Bergsagel P.L., Buadi F.K., Dispenzieri A., Fonseca R., Gertz M.A., Gonsalves W.I., Hayman S.R., Kapoor P. (2017). Therapy for relapsed multiple myeloma: Guidelines from the mayo stratification for myeloma and risk-adapted therapy. Mayo Clin. Proc..

[4] Hideshima T., Richardson P.G., Anderson K.C. (2011). Mechanism of action of proteasome inhibitors and deacetylase inhibitors and the biological basis of synergy in multiple myeloma. Mol. Cancer Ther..

[5] Obeng E.A., Carlson L.M., Gutman D.M., Harrington W.J., Lee K.P., Boise L.H. (2006). Proteasome inhibitors induce a terminal unfolded protein response in multiple myeloma cells. Blood.

[6] Zinszner H., Kuroda M., Wang X., Batchvarova N., Lightfoot R.T., Remotti H., Stevens J.L., Ron D. (1998). Chop is implicated in programmed cell death in response to impaired function of the endoplasmic reticulum. Genes. Dev..

[7] Brewer J.W., Diehl J.A. (2000). Perk mediates cell-cycle exit during the mammalian unfolded protein response. Proc. Natl. Acad. Sci. USA.

[8] Mohty B., El-Cheikh J., Yakoub-Agha I., Moreau P., Harousseau J.L., Mohty M. (2010). Peripheral neuropathy and new treatments for multiple myeloma: Background and practical recommendations. Haematologica.

[9] Cole L.E., Vargo-Gogola T., Roeder R.K. (2016). Targeted delivery to bone and mineral deposits using bisphosphonate ligands. Adv. Drug Deliv. Rev..

[10] Agyin J.K., Santhamma B., Roy S.S. (2013). Design, synthesis, and biological evaluation of bone-targeted proteasome inhibitors for multiple myeloma. Bioorg. Med. Chem. Lett..

[11] Dallas S.L., Garrett I.R., Oyajobi B.O., Dallas M.R., Boyce B.F., Bauss F., Radl J., Mundy G.R. (1999). Ibandronate reduces osteolytic lesions but not tumor burden in a murine model of myeloma bone disease. Blood.

[12] Oyajobi B.O., Munoz S., Kakonen R., Williams P.J., Gupta A., Wideman C.L., Story B., Grubbs B., Armstrong A., Dougall W.C. (2007). Detection of myeloma in skeleton of mice by whole-body optical fluorescence imaging. Mol. Cancer Ther..

[13] Oyajobi B.O., Franchin G., Williams P.J., Pulkrabek D., Gupta A., Munoz S., Grubbs B., Zhao M., Chen D., Sherry B. (2003). Dual effects of macrophage inflammatory protein-1alpha on osteolysis and tumor burden in the murine 5tgm1 model of myeloma bone disease. Blood.

[14] Li X., Sun W., Li J., Wang M., Zhang H., Pei L., Boyce B.F., Wang Z., Xing L. (2017). Clomipramine causes osteoporosis by promoting osteoclastogenesis via e3 ligase itch, which is prevented by zoledronic acid. Sci. Rep..

[15] Zhang H., Hilton M.J., Anolik J.H., Welle S.L., Zhao C., Yao Z., Li X., Wang Z., Boyce B.F., Xing L. (2014). Notch inhibits osteoblast formation in inflammatory arthritis via noncanonical nf-kappab. J. Clin. Investig..

[16] Zhang S., Gangal G., Uludag H. (2007). ‘Magic bullets’ for bone diseases: Progress in rational design of bone-seeking medicinal agents. Chem. Soc. Rev..

[17] Tanaka K.S., Houghton T.J., Kang T., Dietrich E., Delorme D., Ferreira S.S., Caron L., Viens F., Arhin F.F., Sarmiento I. (2008). Bisphosphonated fluoroquinolone esters as osteotropic prodrugs for the prevention of osteomyelitis. Bioorg. Med. Chem..

[18] Terpos E., Heath D.J., Rahemtulla A., Zervas K., Chantry A., Anagnostopoulos A., Pouli A., Katodritou E., Verrou E., Vervessou E.C. (2006). Bortezomib reduces serum dickkopf-1 and receptor activator of nuclear factor-kappab ligand concentrations and normalises indices of bone remodelling in patients with relapsed multiple myeloma. Br. J. Haematol..

[19] Roodman G.D. (2008). Bone building with bortezomib. J. Clin. Investig..

[20] Weathington N.M., Mallampalli R.K. (2014). Emerging therapies targeting the ubiquitin proteasome system in cancer. J. Clin. Investig..

[21] Richardson P.G., Sonneveld P., Schuster M.W., Irwin D., Stadtmauer E.A., Facon T., Harousseau J.L., Ben-Yehuda D., Lonial S., Goldschmidt H. (2005). Bortezomib or high-dose dexamethasone for relapsed multiple myeloma. N. Engl. J. Med..

[22] Bonvini P., Zorzi E., Basso G., Rosolen A. (2007). Bortezomib-mediated 26s proteasome inhibition causes cell-cycle arrest and induces apoptosis in cd-30+ anaplastic large cell lymphoma. Leukemia.

[23] Nawrocki S.T., Carew J.S., Pino M.S., Highshaw R.A., Dunner K., Huang P., Abbruzzese J.L., McConkey D.J. (2005). Bortezomib sensitizes pancreatic cancer cells to endoplasmic reticulum stress-mediated apoptosis. Cancer Res..

[24] Shakespeare W.C., Wang Y., Bohacek R., Keenan T., Sundaramoorthi R., Metcalf C., Dilauro A., Roeloffzen S., Liu S., Saltmarsh J. (2008). Sar of carbon-linked, 2-substituted purines: Synthesis and characterization of ap23451 as a novel bone-targeted inhibitor of src tyrosine kinase with in vivo anti-resorptive activity. Chem. Biol. Drug Des..

[25] Boyce B., Shakespeare W.C., Xing L., Wang Y., Sundaramoorthi R., Keenan T., Metcalf C., Bohacek R., van Schravendijk M.R., Dalgarno D. (2002). Development of a novel bone-targeted src tyrosine kinase inhibitor ap23451 having potental in vitro and in vivo anti-resorptive properties. J. Bone Miner. Res..

[26] Argyriou A.A., Cavaletti G., Bruna J., Kyritsis A.P., Kalofonos H.P. (2014). Bortezomib-induced peripheral neurotoxicity: An update. Arch. Toxicol..

[27] Cavaletti G., Gilardini A., Canta A., Rigamonti L., Rodriguez-Menendez V., Ceresa C., Marmiroli P., Bossi M., Oggioni N., D’Incalci M. (2007). Bortezomib-induced peripheral neurotoxicity: A neurophysiological and pathological study in the rat. Exp. Neurol..

