# Supplementary material for: Synthesis of a Bone-Targeted Bortezomib with In Vivo Anti-Myeloma Effects in Mice
Source: Pharmaceutics. 2018 Sep 10;10(3):154. doi: 10.3390/pharmaceutics10030154 (PMC6161102; doi:10.3390/pharmaceutics10030154)
Supplement: Supplementary file 1 [file pharmaceutics-10-00154-s001.pdf]

pharmaceutics

MDPI

Supplementary materials: Synthesis of a Bone-Targeted Bortezomib with In Vivo Anti-Myeloma Effects in Mice

Hua Wang, Lifeng Xiao, Jianguo Tao, Venkat Srinivasan, Brendan F. Boyce, Frank H. Ebetino, Babatunde O. Oyajobi, Robert K. Boeckman Jr. and Lianping Xing

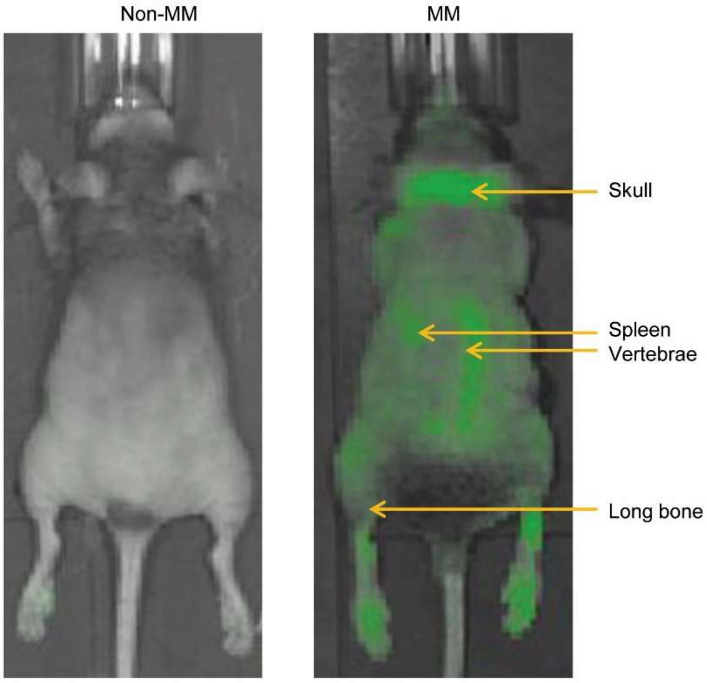

Figure S1. Mouse injected with 5TGM1-GFP cells via the tail vein and subjected to IVIS imaging at 3 weeks post-MM cell inoculation. The IVIS image from a MM mouse (**right**) showing GFP+ MM cells in skull, vertebrae, spleen and legs and from a mouse given no MM cells (**left**).

Table S1. Dosing information of BP-Btz in MM mice.

Group	n	Animals	Drugs	Dose (mg/kg)	Molar dose (μmol/kg)	Frequency	Total dose (μmol/kg)
PBS+Veh	6	PBS	Saline	-	-	Thrice/wk	-
MM+Veh	8	Myeloma cells	Saline	-	-	Thrice/wk	-
MM+Btz	8	myeloma cells	Btz	0.6	1.56	Thrice/wk	9.36
MM+BP-Btz	8	myeloma cells	BP-Btz	1.2	1.68	Thrice/wk	10.08
